# Analyzing the Frequency of Premalignant Lesions and Oral Malignancy in Indian Subjects Attending Outpatient Department From the Low Socioeconomic Group

**DOI:** 10.7759/cureus.42035

**Published:** 2023-07-17

**Authors:** Taruna Taruna, Yatendra P Singh, Renu Waghmare, Abhishek Sinha, Leena Priya, Varsha Sharma, Uday B Singh

**Affiliations:** 1 Public Health Dentistry, Patna Medical College and Hospital, Patna, IND; 2 Otorhinolaryngology, Saraswati Medical College and Hospital, Unnao, IND; 3 Preventive Social Medicine, Nandkumar Singh Chouhan Government Medical College, Khandwa, IND; 4 Dentistry, Patna Medical College and Hospital, Patna, IND; 5 Oral Medicine and Radiology, Patna Medical College and Hospital, Patna, IND; 6 Periodontology, Bhabha College of Dental Sciences, Bhopal, IND; 7 Oral and Maxillofacial Surgery, Recarve Maxillofacial Centre and Dental Studio, Delhi, IND

**Keywords:** survey, precancerous lesions, oral cancer, cancer screening, cancer detection

## Abstract

Background: Oral cancer is a rapidly growing disease among Indian subjects mainly in the low socioeconomic group. Socially and economically marginalized subjects are at high risk for oral cancer because of smoke and smokeless tobacco consumption.

Aim: To evaluate the prevalence of precancerous lesions and oral cancer and evaluate tobacco as a causative factor in Indian subjects visiting the outpatient department of the institute.

Methods: Around 658 subjects were analyzed for frequency of premalignant lesions and oral malignancy in Indian subjects from the low socioeconomic group. Patients visited for pain, burning, or ulceration in the oral cavity were clinically assessed for any tissue growths, leathery alterations, ulcerative changes, and white or red lesions in the oral cavity.

Results: The overall prevalence of smokeless and smoking tobacco was 78.8% (n=518) and 65.2% (n=429) respectively in the present study. Around 39.8% (n=262) of samples were stained positive for precancerous and cancerous lesions of the oral cavity. The highest number of positive samples were from buccal mucosa with 36.2% (n=238) subjects and 6.1% (n=40) for labial mucosa.

Conclusions: Oral cancer is highly prevalent in Indian subjects owing to high tobacco consumption rates and habits warranting the cessation center a priority. Also, early detection and screening are vital to attaining better outcomes. More tobacco cessation centers are needed to stop the habit and early diagnosis will prevent dysplastic changes.

## Introduction

India is the major contributor to the burden of oral cancer with the highest incidence reported in the World as mentioned by Gupta and Ray [1]. Occurrence of oral cancer can be attributed to lifestyle-related habits including the use of a paste of slaked lime and areca nut, betel leaf, flavoring agents having tobacco, coloring agents having tobacco, sweetening agents having tobacco, catechu, lime, areca nut, pan masala, tobacco, alcohol, and/or smoking [1] - these above practices are more common in Indian population. In the Asian population, the most common form of tobacco used is in a smokeless form. Along with the traditional agents like tooth powder having tobacco, lime with tobacco, pan masala, i.e. adding more spices, flavors, and lime with betel leaf, betel quid, and areca nuts. The use of newer products containing tobacco is increasing not only in male subjects, but also in dental students, medical students, teenagers, children, and female subjects of reproductive age [2-3]. 

Oral cancer is a major healthcare concern globally including in India where nearly 30% of cancers are contributed by oral cancer. The difference is seen in the prevalence and incidence of oral cancer based on region owing to different risk factors prevalent. Late diagnosis leads to high mortality rates in affected subjects [4]. Early detection can help in a better-improved survival rate and treatment outcomes. But those from low socioeconomic backgrounds are more likely to develop oral cancer, and they have less access to early treatment, which leads to delayed diagnosis and management and low survival rates [5].

Previous literature data report a high prevalence, incidence, and concern for oral cancer at different time intervals. A large Indian population is from a poor background with associated issues of social discrimination of different habits, poor healthcare, communication issues, illiteracy, and/or poverty which further limit their access to oral healthcare [6].

Alcohol intake and tobacco consumption constitute the most common etiologic factors for oral cancer seen in >90% of the affected subjects. In subjects taking tobacco, Indian subjects from the low socioeconomic background are seen to use smoking and smokeless tobacco in high quantities as reported by previous literature data from Indian studies. It is also reported that for subjects of age >15 years from low socioeconomic backgrounds, more than 50% used tobacco in either smoking like bidi, cigarette, cigar, or smokeless forms like pan masala, gutkha, and betel quid. Also, medium nicotine dependency was seen in subjects using smokeless or smoke tobacco form [7].

These data point to the high prevalence of oral precancerous lesions and cancer in the regional Indian population. However, the data concerning this issue is scarce in the literature [8]. Hence, the present study aimed to evaluate the prevalence of precancerous lesions and oral cancer in Indian subjects from the low socioeconomic group from an urban population which indicates a small scale of different populations of India. 

## Materials and methods

The present cross-sectional descriptive clinical study was aimed to evaluate the prevalence of precancerous lesions and oral cancer in Indian subjects from low socioeconomic groups mostly with population from labor, people depending on daily wedges, and low economic/unemployed groups. The study was done after clearance was given from the Institutional Ethical Committee concerned. The study population was recruited from the Outpatient Department of Oral Medicine and Radiology of the Institute after the subjects signed a written informed consent [PMCH/2023/136].

The inclusion criteria were subjects of age 15-75 years of age, with tobacco chewing or smoking habits from either gender, and signed the written consent. Subjects who were immunocompromised, unwilling to participate in the trial, and receiving treatment for oral cancer or precancerous lesions were excluded from the study. 

Based on previously published or preclinical studies, estimating the effect of magnitude, demographic information was gathered for each subject in the Oral Medicine and Radiology Outpatient Department. The data about residential address, birth date, and age gathered were confirmed with the ID of the subject (Aadhar card). The sample size for the study was 658 subjects from both genders. Sociodemographic data, including place of birth, age, gender, and level of education; self-reported medical history; family history of oral cancer; dietary and physical activity preferences; cigarette and alcohol use; and oral symptoms. Information on alcohol intake, cigarette use, and oral cancer in the family was taken into account for the analysis. After recording the demographic data, a clinical examination of the oral cavity was done for all the participants by two oral examiner experts in the field.

Among these two examiners, one was from the Department of Oral Medicine and the other from the Department of Oral Pathology. The clinical examination was done through the visual inspection of the oral cavity. The oral cavity was inspected for any tissue growths, hyperkeratotic changes in the oral cavity, ulcerative alterations, and/or red or white lesions. The WHO criteria of 1997, were used to diagnose precancerous lesions and conditions. The subjects suspected to have potential premalignant lesions were identified and sent for counseling at the tobacco cessation center to make subjects quit the habit. We considered it a reference Indian population, not central India or overall India.

Variables studied were tissue growths, leathery alterations, ulcerative changes, and white or red lesions in the oral cavity assessed under adequate light. 

The data obtained were assessed statistically to evaluate the prevalence which was described in terms of percentage. The results were expressed in mean and standard deviations and percentages and numbers. The Chi-square test was used to compare lesion prevalence in females and males with a significance level of p < 0.05.

## Results

The study included 658 subjects where there were 50.45% (n=332) males and 49.54% (n=326) females. The mean age of female and male subjects was 37.4 ± 2.22 years and 40.2 ± 2.24 years, respectively. The overall prevalence of smokeless and smoking tobacco was 78.8% (n=518) and 65.2% (n=429) respectively in the present study. The type of chewed tobacco/pan masala in females and either smoked or chewed tobacco in males. Variables assessed were tissue growths, leathery alterations, ulcerative changes, and white or red lesions in the oral cavity were assessed under adequate light. 

For site-wise distribution, any white lesion, red lesion, and growth/ulceration were seen in the oropharynx, gingiva, palate, floor of the mouth, and vestibule to correlate for hyperkeratotic and dysplastic changes. White lesions and red lesions each were seen in 0.3% (n=2), 0.60% (n=4), and 41.64% (n=274) subjects respectively in tongue and labial mucosa. White and red lesions were seen in 41.64% (n=274) and 9.11% (n=60) subjects, respectively. Ulceration/growth was seen in 0.3% (n=2) of study subjects. Leathery changes were seen in 19.14% (n=126), 16.10% (n=106), 6.38% (n=42), 0.3% (n=2), 11.24% (n=74), 1.21% (n=8), 16.41% (n=108), and 28.26% (n=186) study subjects respectively as shown in Table 1.

**Table 1:** Site-wise distribution of oral lesions in study subjects.

Sr. no.	Site	Leathery changes n (%)	Growth/ulceration n (%)	Red lesions n (%)	White lesions n (%)
1	Oropharynx	126 (19.14)	0	0	0
2	Gingiva	106 (16.10)	0	0	0
3	Palate	42 (6.38)	0	0	0
4	The floor of the mouth	2 (0.3)	0	0	0
5	Vestibule	74 (11.24)	0	0	0
6	Tongue	8 (1.21)	0	2 (0.3)	2 (0.3)
7	Labial mucosa	108 (16.41)	0	4 (0.60)	4 (0.60)
8	Buccal mucosa	186 (28.26)	2 (0.3)	60 (9.11)	274 (41.64)
9	Total	652 (99.08)	2 (0.3)	66 (10.03)	280 (42.55)

Gender was considered a variable, in male subjects; the prevalence of precancerous and cancerous lesions was 57.2% (n=182) subjects, and in females, it was 42.7% (n=136) subjects. The difference was statistically non-significant with p=0.07 (Table 2).

**Table 2:** Gender-wise distribution of oral lesions in study subjects.

Gender	n (%)	p-Value
Males	146 (22.18)	0.07
Females	116 (17.62)	

Though the results were based on clinical features, toluidine blue staining was used to differentiate dysplastic tissues. In the present study, 39.8% (n=262) of samples were stained positive for precancerous and cancerous lesions of the oral cavity. The positive staining was seen for 44% (n=289) males and 35.6% (n=234) females as shown in Table 3. Further samples were sent for biopsy to detect any malignancy confirmation.

**Table 3:** Site and gender-wise distribution of oral lesion in study subjects after toluidine.

Gender	Oropharynx n (%)	Gingiva n (%)	Palate n (%)	The floor of mouth n (%)	Vestibule n (%)	Tongue n (%)	Labial mucosa n (%)	Buccal mucosa n (%)
Males	4 (0.60)	6 (0.91)	2 (0.3)	0	6 (0.91)	2 (0.3)	28 (4.25)	136 (20.66)
Females	4 (0.60)	8 (1.21)	4 (0.60)	2 (0.3)	10 (1.51)	2 (0.3)	12 (1.82)	102 (15.50)

The highest number of positive samples were from buccal mucosa with 36.2% (n=238) subjects and 6.1% (n=40) for labial mucosa as shown in Table 4.

**Table 4:** Site-wise distribution of oral lesions in study subjects after toluidine blue stain.

S. no.	Site	Number (n)	Percentage (%)
1	Oropharynx	8	1.21
2	Gingiva	14	2.12
3	Palate	6	0.91
4	The floor of the mouth	2	0.3
5	Vestibule	16	2.43
6	Tongue	4	0.60
7	Labial mucosa	40	6.07
8	Buccal mucosa	238	36.28

## Discussion

In the majority of developing countries, a large population is from a lower socioeconomic background with a considerable population belonging to a socially marginalized group -- population included mostly labor, people depending on daily wedges, and low economic/unemployed group. This population has limited access to healthcare services and is underprivileged. These subjects are prone to develop deleterious habits including tobacco consumption leading to a high prevalence of oral lesions as suggested by Srivastava et al. [9] in 2020. Subjects from lower socioeconomic status are at higher risk of developing oral cancer by 1.8 times as reported by Warnakulasuriya [10] in 2009.

Concerning the intake of tobacco and related habits, the study results reported a high prevalence of precancerous and cancerous lesions -- 57.2% (n=182) male subjects and 42.7% (n=136) female subjects. The difference was statistically non-significant with p=0.07. This tobacco intake was peer-induced and culture-based. As the mean age of habit was 14.5 ± 2.1 years, the habits started early in life and sustained longer, and passed from generation to generation as reported by Khanna [11] in 2012 and Palliyal [12] in 2019. Other attributing factors were confronting hunger, poverty, and unemployment. 

The study results showed that following toluidine blue staining, 39.8% (n=262) samples were stained positive for precancerous and cancerous lesions of the oral cavity. The positive staining was seen for 44% (n=289) males and 35.6% (n=234) females. The highest number of positive samples were from buccal mucosa with 36.2% (n=238) subjects and 6.1% (n=40) for labial mucosa. The difference was statistically non-significant with p=0.07. These results coincided with the studies of Vinay et al. [13] in 2014 where authors reported a significantly higher prevalence of oral precancerous lesions in male subjects compared to females and studies of Srivastava et al. [9] in 2020 where a high prevalence of leukoplakia and oral submucous fibrosis (OSMF) was seen in male subjects.

The majority of the lesions reported in the study were in the buccal mucosa. These results were against the findings of Pullishery [14] in 2018 where authors reported maximum lesions in the tongue in 45.5% of subjects followed by 39% lesions in the buccal mucosa. The high prevalence was due to tobacco-related habits.

The overall prevalence of smokeless and smoking tobacco was 78.8% (n=518) and 65.2% (n=429) respectively in the present study. Other studies as by Khanna et al. [11] in 2012 reported a 10.3% prevalence of leukemia in their studies; Valsan et al. [15] in 2016 reported 4.2% of subjects having oral mucosal lesions, whereas Francis [16] in 2020 reported a 52% prevalence of oral mucosal lesions.

Limitations

The present study had a few limitations including a smaller sample size, single-institute assessment, shorter monitoring period, and limited laboratory resources. These limitations can lead to inappropriate results and underestimation of the actual burden of oral cancer in the population defined in the present study.

## Conclusions

The study aimed to assess the oral precancerous and cancerous lesions in low socioeconomic groups. A high prevalence of oral lesions was seen in both genders in subjects with low socioeconomic backgrounds. The study suggests the initiation of nationwide programs and tobacco cessation centers to prevent the outbreak. Also, early detection and screening programs are needed to decrease the disease burden.
